# Comparison of recovery after sugammadex or neostigmine reversal of rocuronium in geriatric patients undergoing spine surgery: a randomized controlled trial

**DOI:** 10.3325/cmj.2021.62.606

**Published:** 2021-12

**Authors:** Boris Mraovic, Noah J. Timko, Theodore J. Choma

**Affiliations:** 1 Department of Anesthesiology and Perioperative Medicine, University of Missouri Columbia, Columbia, MO, USA; 2 Department of Orthopaedic Surgery, University of Missouri Columbia, Columbia, MO, USA

## Abstract

**Aim:**

To evaluate the effect of sugammadex compared with neostigmine on speed and quality of recovery after rocuronium neuromuscular blockade (NMB) in geriatric patients undergoing posterior lumbar spine surgery.

**Methods:**

This randomized controlled study at a tertiary academic medical center involved 40 patients (age ≥65 years, ASA PS II/III) scheduled for elective surgery under general anesthesia. Patients were randomized to sugammadex or neostigmine for reversal of moderate NMB with rocuronium. The primary outcome was recovery time from NMB after surgery to a train-of-four (TOF) ratio ≥0.9 measured at the adductor pollicis (TOF-Watch^®^ SX). Secondary outcomes included hemodynamic change after administration of reversal agent (heart rate, blood pressure, dysrhythmia), time to extubation, pain medication requirement, time to first ambulation, and length of postanesthesia care unit (PACU) and total hospital stay.

**Results:**

Sugammadex (4 ± 2.2 min) compared with neostigmine reversal (26.3 ± 17.5 min) was on average 22 min faster (95% CI 14.1-30.5; *P* <0.001) with less variability (range 2-11 min vs 5-72 min). The groups significantly differed in time for tracheal extubation, response to verbal commands (open eyes, squeeze hand, lift head), and operating room exit. However, they had similar PACU stay, time to first ambulation, total hospital stay, postoperative pain, and opioid use. Sugammadex had less hemodynamic variability than neostigmine. No patient developed treatment-emergent dysrhythmias.

**Conclusion:**

Sugammadex reversal significantly hastened NMB recovery compared with neostigmine reversal in geriatric patients. It significantly decreased operating room time but not PACU time or hospital stay.

**ClinicalTrial.gov identification number:**

NCT03112993

Spine surgery is one of the most common surgeries in the United States (1). Although spine surgeries are often performed in middle-age patients, they are becoming more frequent and can have greater complexity in geriatric patients (aged ≥65 years) (1). The elderly population is the fastest growing segment of the American population, and the number of spine surgeries in this population is expected to considerably increase.

Lumbar spine surgery is performed in the prone position for decompressive laminectomy and fusion, and neuromuscular blocking agents (NMBA) are used to improve operating conditions (2). Rocuronium is a commonly used NMBA for anesthesia induction and maintenance, but its duration of action can highly vary in the geriatric patient. At the end of these surgeries, NMBA are reversed immediately after returning patients supine to avoid bucking, coughing, and accidental extubation in the prone position. Neostigmine reversal of NMBA has a long history of usage but it could be prolonged and unpredictable in elderly patients (3,4). Reversal with sugammadex is usually faster and more predictable than that with neostigmine, but comparison studies in elderly patients undergoing spine surgery in prone position are sparse (5).

In this study, we compared the speed and quality of recovery after reversal of neuromuscular blockade (NMB) by rocuronium with sugammadex or neostigmine in geriatric patients undergoing elective posterior lumbar spine surgery in the prone position.

## PATIENTS AND METHODS

### Study design

This prospective, double-blind, randomized controlled trial was approved by the University of Missouri Institutional Review Board (2008066) on March 22, 2017 and registered at *clinicaltrials.gov* (NCT03112993) on April 13, 2017. All procedures were performed in a single-center tertiary academic medical hospital. Patients were screened between May 2017 and August 2018. The study was conducted in accordance with the principles of the Declaration of Helsinki. Written informed consent was obtained from all patients before any study procedures were performed.

## Participants

Patients 65 years or older with American Society of Anesthesiology physical status (ASA PS) I to III scheduled for elective posterior lumbar spine surgery with either decompression laminectomy or fusion under general anesthesia in the prone position were eligible for the study. The exclusion criteria were inability to provide written informed consent, allergy to medications or anesthetic agents used in the study, neuromuscular disorders, liver disease, renal disease or serum creatinine ≥2 mg/dL, and family history of malignant hyperthermia.

### Randomization and blinding

At the time of NMB reversal, patients were randomized by using random permuted block either to receive sugammadex (Bridion, Merck and Co., Inc., Kenilworth, NJ, USA) 2 mg/kg IV or neostigmine (Bloxiverz, Avadel Pharmaceuticals, Chesterfield, MO, USA) 50 µg/kg IV not to exceed 5 mg with glycopyrrolate, 10 µg/kg IV not to exceed 1 mg, and further stratified by surgical approach (decompression or fusion). Before the start of patient enrollment, a biostatistician created the randomization list and wrote group assignments on notecards, which were placed within sealed tamper-resistant security envelopes (identified externally by unique study identification number). On the day of surgery after patient randomization assignment, an anesthesia provider not involved in the care of the study patients was provided the envelope corresponding to the assigned unique identification number on the randomization list. The anesthesia provider prepared the reversal in a syringe normalized with saline to 10 mL for all patients. The 10-mL clear solution could not be recognized by clinical and research personnel and blinded what reversal agent patients were receiving. Blinded research personnel collected all patients’ data.

### Management of anesthesia

After obtaining written informed consent, all patients received the same standardized general anesthesia with standard anesthesia monitoring. Anesthesia was induced with lidocaine 1 mg/kg IV, propofol 1 mg/kg IV, fentanyl 2 µg/kg IV, and rocuronium 0.6 mg/kg IV. Additional propofol or fentanyl was given at the discretion of anesthesia providers. Anesthesia was maintained with sevoflurane ~ 1 minimum alveolar concentration (MAC) in oxygen/air (fraction of inspired oxygen 0.6) at 2 L/min adjusted to maintain a bispectral index level of 40-60 (BIS Quatro sensor, Covidien, Mansfield, MA, USA). NMB was maintained with rocuronium infusion, 3 µg/kg/min started at the first reappearance of 1 twitch train-of-four (TOF) and titrated to maintain 2 twitches TOF on the TOF-Watch® SX (Organon, Inc., West Orange, NJ, USA) monitor throughout the procedure. TOF (TOF stimulation every 15 sec) was monitored at the adductor pollicis. The TOF-Watch® SX was calibrated after induction and before the first dose of rocuronium. At the surgical skin closure, sevoflurane was decreased to 0.5 MAC with the same fresh gas flow. At the end of surgery, the rocuronium infusion was stopped, and 100% oxygen 10 L/min was turned on just before turning the patient from the prone to supine position for emergence and extubation.

The reversal agent was given when the patient was turned back to the supine position immediately following baseline hemodynamic measure collection and confirmation of appropriate body temperature. Hemodynamics (blood pressure and heart rate) were recorded every minute and TOF until TOF ratio (TOFR) was ≥0.9 for 3 consecutive measures. Patients were monitored for dysrhythmias after reversal agent administration. If patients were waking up, fighting the ventilator, coughing, or bucking before they reached TOFR≥0.9, inhaled 50% N_2_O was delivered to avoid recall and to facilitate appropriate monitoring of NMB at the adductor pollicis. Patients were required to have a TOFR≥0.9 and meet the clinical criteria for NMB reversal (open eyes and follow verbal commands to squeeze hand/lift head) before extubation.

All patients had body temperature maintained (core temperature ≥35 °C) during surgery with upper body forced-air patient warmer (3M Bair Hugger System, St. Paul, MN, USA), and after surgery by warm blankets. If NMB emergence was prolonged (≥15 min), body temperature was additionally maintained by using full body forced-air patient warmer (3M Bair Hugger System) adhering to the requirement of TOF-Watch® SX. Hydromorphone 0.5 mg IV was given after fusion procedures at the end of surgery, and a hydromorphone patient-controlled analgesia pump was started in the postanesthesia care unit (PACU). Fentanyl 25 µg IV was given PRN for decompression procedures. A verbal 11-point scale (0 “no pain” through 10 “worst pain imaginable”) was used to assess postoperative pain.

### Outcome measures

The primary outcome was the time from administration of a NMBA reversal agent to return of three consecutive measures of the TOFR≥0.9. Secondary outcome measures were time to extubation, difference from baseline (<2 min prior to reversal agent administration) of hemodynamics (heart rate, blood pressure, dysrhythmias) after administration of reversal agent, time to operating room (OR) exit, PACU readiness time (Aldrete scoring system; target score 9 or 10 [range: 0-10]; first assessed 15 min after PACU entry with repeat measures every 15 min) as well as PACU discharge time, pain medication requirement, time to first ambulation, and length of hospital stay. Patients were also assessed with a questionnaire for postoperative satisfaction of anesthesia care on postoperative day 1 (6).

### Statistical analysis

Sample size calculation for the primary endpoint (TOFR≥0.9) in geriatric patients used 2.9 ± 1.6 min for sugammadex reversal (3). Since neostigmine reversal could vary from 6 min to over 20 min (7-9), 20 patients per patient group were needed assuming a standard deviation of 50% to have 90% power to detect a significant difference between groups in mean times of as little as 6 min (alpha <0.05). Additionally, this sample size would provide a power level of 0.8 and alpha 0.05 to detect a difference of 12 min or greater in mean time spent in PACU between study arms.

Data were analyzed with the χ^2^ and *t* tests as appropriate. The Wilcoxon rank-sum test was used to compare the two groups with respect to continuous baseline characteristics. The Fisher exact test was used to compare nominal-scale variables. For the surgical outcomes, a stratified version of the Wilcoxon rank-sum test was used with procedure type (fusion or decompression) defining the strata. No correction was made for multiple testing. *P* < 0.05 was considered significant. Statistical analysis was performed with SAS for Microsoft Windows, version 9.2 (SAS Institute, Inc., Cary, NC, USA).

## RESULTS

**Figure 1:**
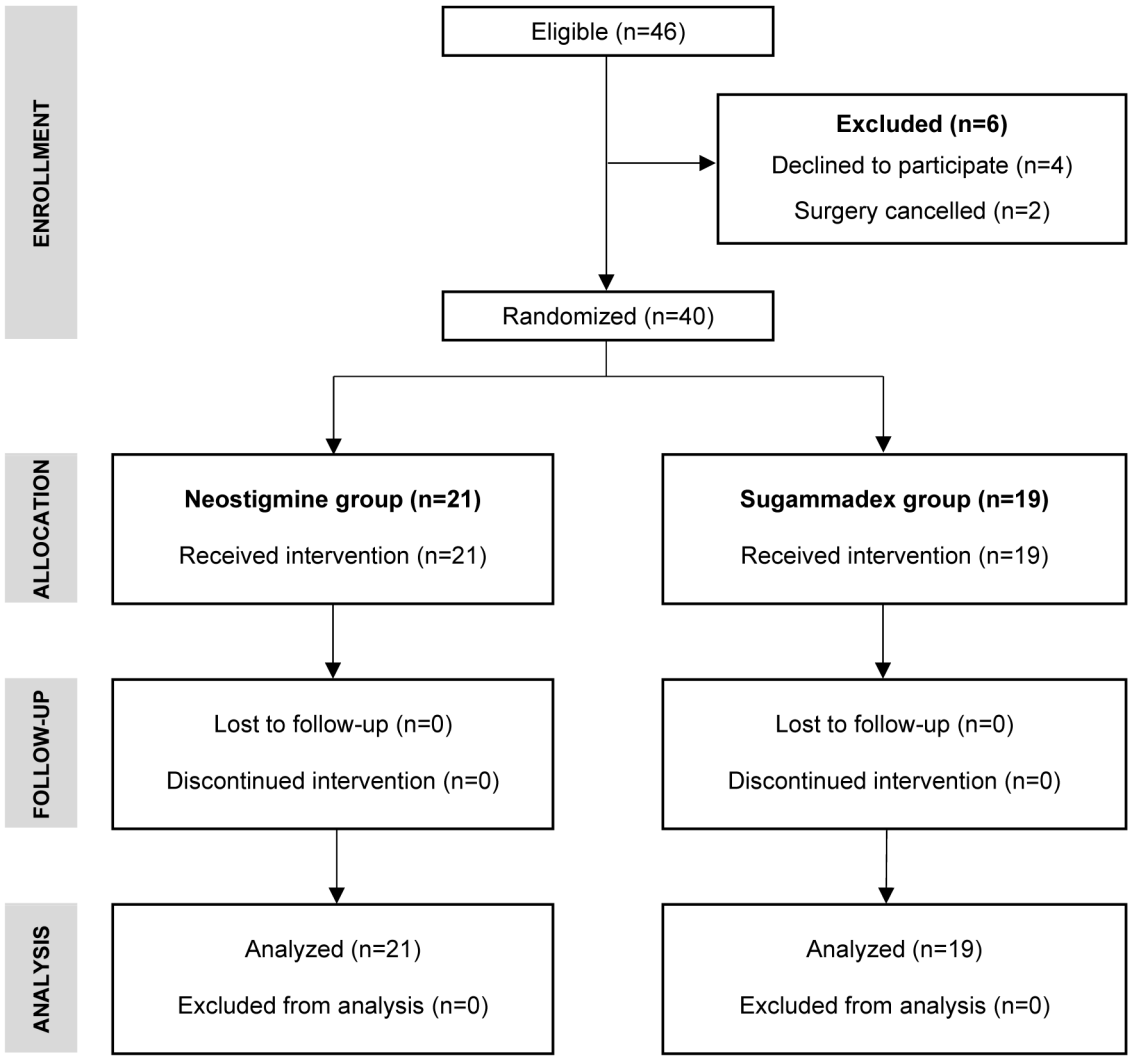
Flow diagram (CONSORT) of spine surgery patients through the trial.

Forty-six patients were assessed and 40 completed the study (Figure 1). The groups did not differ in demographic data, except that the neostigmine group had longer duration of surgery and anesthesia, on average 21 min and 35 min, respectively (Table 1). Intraoperative medication administration was similar between study groups, except that the neostigmine group received on average 45 mg more rocuronium and about 200 mL more crystalloid because of longer duration of surgeries (Table 2). The reversal agents were given about 5 min after the end of surgery in both groups (Table 3). Just before NMB reversal, the TOF was 2 twitches in all patients in both groups. In the sugammadex group, TOFR≥0.9 was reached on average 22 min faster (95% CI 14.1-30.5; *P* = <0.001) than in the neostigmine group; 4 min vs 26 min, respectively (Table 3). The sugammadex group had significantly faster recovery in the OR after NMB reversal administration (extubation, following verbal commands, opening eyes, time to OR exit). However, recovery times in the postoperative period did not differ, as well as stay in PACU and time to the first ambulation (Table 3). Length of stay in hospital was on average 1.3 days (95% CI, 0.6-3.1, *P* = 0.098) longer in the neostigmine group, but the difference was not significant (Table 3). PACU opioid consumption and the use of ondansetron or promethazine were similar in both groups, as were the postoperative pain scores (Table 4).

**Table 1:** Patient demographics and baseline characteristics of the surgical procedure. Data are means (standard deviations) or number (%) of patients

	Neostigmine (n = 21)	Sugammadex (n = 19)
Age, years	71.2 (4.8)	70.4 (4.7)
Weight, kg	94.5 (14.7)	91.7 (17.9)
Height, cm	173.9 (9.5)	170.1 (9.4)
Body mass index, kg · m^−2^	31.4 (5.2)	31.8 (6.4)
Sex		
male	17 (81%)	10 (53%)
female	4 (19%)	9 (47%)
Race		
White	20 (95%)	18 (95%)
Black	1 (5%)	1 (5%)
Surgery		
decompression	11 (52%)	8 (42%)
fusion	10 (48%)	11 (58%)
American Society of Anesthesiologists physical status classification		
II	5 (24%)	8 (42%)
III	16 (76%)	11 (58%)
Intraoperative blood transfusion	2 (9%)	1 (5%)
Blood loss, mL	223 (280)	159 (191)
Duration of surgery, min	196 (96)	175 (125)
Duration of anesthesia, min	253 (111)	218 (128)

**Table 2:** Intraoperative medications. Data are means (standard deviations)

	Neostigmine (n = 21)	Sugammadex (n = 19)	*P*
Propofol, mg	114 (32)	104 (37)	0.254
Lidocaine, mg	61 (17)	64 (16)	0.776
Fentanyl, µg	153 (95)	166 (96)	0.535
Rocuronium, mg	128 (91)	83 (50)	0.018
Dexamethasone, mg	7.1 (3.8)	6.5 (3.5)	0.330
Ondansetron, mg	4 (1.3)	3.8 (0.9)	0.618
Hydromorphone, mg	0.8 (0.7)	0.6 (0.6)	0.171
Crystalloid, mL	1733 (702)	1503 (775)	0.036

**Table 3:** Postoperative patient recovery measures. Data are means (standard deviations) unless otherwise stated*

	Neostigmine (n = 21)	Sugammadex (n = 19)	Difference (95% CI)	*P*
TOF ratio ≥0.9, min	26.3 (17.5)	4.0 (2.2)	22.3 (14.1 to 30.5)	<0.001
Reversal administration, min	4.8 (1.4)	5.5 (2.1)	-0.7 (-0.5 to 1.8)	0.219
Extubation, min	23.6 (16.7)	5.3 (2.5)	18.3 (10.4 to 26.1)	<0.001
Follow verbal commands, min	23.1 (16.7)	5.6 (4.3)	17.6 (9.6 to 25.5)	<0.001
Open eyes, min	22.6 (16.7)	5.6 (4.3)	16.9 (9 to 24.9)	<0.001
Operating room exit, min	31 (17.5)	13.7 (3.5)	17.3 (9 to 25.6)	<0.001
PACU readiness, min	54.6 (21.8)	50.8 (18.4)	3.8 (-9.1 to 16.8)	0.354
Time in PACU, min	85.3 (29.5)	81.4 (16.5)	3.9 (-11.6 to 19.4)	0.951
First ambulation, hours	17.8 (11.1)	17.4 (14.6)	0.4 (-7.8 to 8.7)	0.259
Hospital stay, days	4.4 (3.2)	3.1 (2.6)	1.3 (-0.6 to 3.1)	0.098

*Abbreviations: CI – confidence interval; TOF – train-of-four; reversal administration – end of surgery to administration of reversal agent; operating room exit – end of surgery to operating room exit; PACU – postanesthesia care unit; PACU readiness – patient readiness to leave PACU determined by Aldrete scoring scale; first ambulation – time from end of surgery to first patient ambulation.

**Table 4:** Postanesthesia care unit pain and nausea/vomiting treatment. Data are means (standard deviations) or number (%) of patients unless otherwise stated*

	Neostigmine (n = 21)	Sugammadex (n = 19)	Difference (95% CI)	*P*
Opioid consumption, MME	24 (33.2)	17.6 (15.2)	6.3 (-10.5 to 23.2)	0.566
NRS score	3.4 (2.4)	4.7 (2.3)	-1.3 (-2.7 to 0.2)	0.095
Fentanyl	15 (71%)	14 (74%)	-2.3% (-0.3 to 0.2)	1.000
Hydromorphone	14 (67%)	11 (58%)	8.7% (-0.2 to 0.4)	0.745
Ondansetron	5 (24%)	2 (11%)	13.3% (-0.1 to 0.4)	0.412
Promethazine	4 (19%)	1 (5%)	13.8% (-0.1 to 0.3)	0.345

*Abbreviations: CI – confidence interval; MME – morphine milligram equivalents; NRS – numeric rating scale (11-point).

After reversal agent administration, no patients experienced significant intraoperative bradycardia or heart dysrhythmias. Administration of neostigmine increased heart rate on average more than 10 beats per minute at 2 min, 3 min, and 4 min after NMB reversal, but in the sugammadex group heart rate stayed stable (Table 5). Heart rate measures were not compared further past 4 min as the number of patients experiencing full NMB reversal (TOFR≥0.9) by sugammadex reduced the size of the comparative group. After NMB reversal, patient satisfaction was similar between the groups, with no measure reaching a significant difference. The results were also similar between the groups when fusion and laminectomy surgeries were analyzed separately.

**Table 5:** Heart rate change after neuromuscular blockade reversal agent administration. Data are means (standard deviations) or numbers unless otherwise stated*

	Time, min*	Patients	Heart rate, beats per minute^†^	*P^‡^*	*P* ^§^
Neostigmine	1	21	6.4 (9.9)	0.004	0.170
	2	21	11.3 (11.3)	<0.001	0.001
	3	21	12.5 (11.6)	<0.001	<0.001
	4	21	13.3 (12.1)	<0.001	<0.001
Sugammadex	1	19	2.1 (5.3)	0.090	
	2	19	1.3 (6.4)	0.371	
	3	15	1.1 (7.4)	0.395	
	4	8	-1.4 (9.0)	0.408	

*Minutes after reversal agent administration.

^†^Heart rate change from baseline after reversal agent administration.

‡Comparison of heart rate change from time of reversal agent administration.

§Comparison of neostigmine to sugammadex at each time.

## DISCUSSION

This prospective, randomized controlled, double-blind study, showed on average 22 min faster reversal of rocuronium NMB with sugammadex compared with neostigmine in geriatric patients undergoing elective lumbar spine surgery in the prone position. Reversal time for neostigmine had high variability (5 to 72 min), while that for sugammadex had low variability (2 to 11 min).

Our data are consistent with a study by Blobner et al (7), which also showed high variability and longer surgery duration with neostigmine reversal (median 18.4 min, range 3 to 68 min) but not with sugammadex reversal (median 1.4 min, range 1 to 5 min) in middle-age patients (mean 51 ± 16 years and 48 ± 14 years, respectively) (7). However, the reversals in our study were about 5 min longer, suggesting more time was needed to reach TOFR≥0.9 to avoid residual NMB, respiratory complication, and increased PACU and hospital length of stay (4). Indeed, in our study once patients reached TOFR of 0.9 at emergence from anesthesia, their OR time, PACU recovery, time to first ambulation and hospital stay were similar.

In our study, duration of NMB from the initial intubating rocuronium dose was highly variable, similar to previous reports. However, it was much shorter (median 20 min; range 4 to 72 min) than in a study on patients older than 60 years undergoing elective surgery (median of 63.1 min; range 33 to 119 min) (3). This is probably because the majority of our spine surgery patients received gabapentin, which shortens the duration of action for NMBAs. In addition, different study methods would contribute to longer rocuronium NMB duration in the Arain et al study (3), which measured return to 25% twitch height, whereas we assessed the first reappearance of TOF. Since the clearance and half-life of rocuronium is prolonged with aging, which results in a highly variable duration of action and time to reversal (3), we chose to maintain NMB by rocuronium infusion started at the first reappearance of TOF after the initial intubating dose. The neostigmine group received on average 45 mg of rocuronium more than the sugammadex group probably because they had on average 21 min longer surgery, and the rocuronium infusion was titrated to TOF and not only to patients’ weight. In this way, we avoided frequent rocuronium redosing while maintaining a consistent level of NMB (TOF of 2 twitches) during the procedure. This allowed all patients to have predictable TOF of 2 twitches at reversal administration with minimal influence on the total amount of rocuronium given. Moreover, rocuronium infusion was easy to titrate in our elderly patients, and muscle relaxation remained stable throughout the surgery with good surgeon satisfaction. In spine surgery, NMBA relaxation of the lumbar paraspinal musculature makes lateral retraction easier for the surgeons. Deeper NMB showed better operative conditions, lower inspiratory pressures, and less blood loss (2,9).

During spine surgery, anesthesia and NMB depth are maintained until the end of the procedure. In prone lumbar spine surgery, the goal is emergence and extubation as soon as patients are returned to the supine position. To avoid prolonged emergence, the anesthetic level is decreased and/or NMB is reversed at the start of the surgical wound closure. However, doing both at the same time before the patient is returned to the supine position may not be safe because the surgical closure could last longer than expected. Patients could start to move, buck, cough, or fight the ventilator and have inadvertent tracheal extubation while prone. Reversing NMB in the prone position but keeping anesthetic depth deep could lead to prolonged emergence, especially in the elderly population because of increased sensitivity to volatile anesthetics. Stopping only the anesthetic and reversing NMB after turning back to supine could increase the risk of awareness, and reversal could also be prolonged if neostigmine is used. In our study, the median time from decreasing sevoflurane to 0.5 MAC (time of wound closure) to stopping sevoflurane at the end of the surgery (shortly before returning patent supine) was 22 min (range 2 to 53 min). Therefore, we showed that reducing anesthetic level to 0.5 MAC sevoflurane at the time of prone spine surgery closure and reversing NMB with sugammadex after turning the patient to the supine position was safe, fast, and predictable. Indeed, all our patients reversed with sugammadex left the OR after reversal was given within 15 min (median 8 min, range 4 to 15 min). This could save OR time by improving turnover (end of the surgery to the start of the next case) by over 1 hour in a typical OR with three turnovers a day.

To counteract its cardiac muscarinic effects, neostigmine must be administered with an anticholinergic agent such as glycopyrrolate. The incidence of cardiac dysrhythmias in elderly patients who received neostigmine and glycopyrrolate for NMB reversal was up to 16% (10). We did not observe cardiac dysthymias, but the use of neostigmine and glycopyrrolate increased heart rate compared with baseline and sugammadex but did not change blood pressure. After neostigmine administration, no intervention or medication were needed to correct tachycardia. Sugammadex did not change heart rate or blood pressure with bradycardias in any patient.

Limitations to our study include single-center setting with one type of surgery. Therefore, results could be interpreted only in geriatric patients undergoing prone-position lumbar spine surgery. Our study showed no significant difference in the length of hospital stay but the hospital stay was on average 1.3 days longer in the neostigmine group, with median duration 2.5 days longer (neostigmine group, 4.7 days, range 0.4 to 13.9 days; sugammadex group, 2.2 days, range 0.1 to 9.1 days). Future studies in this study population would require a larger sample size with 111 patients in each group at a power level 0.8 and alpha 0.05 to show a difference between groups in the length of hospital stay.

In summary, this randomized controlled double-blind clinical trial showed that reversal of NMB with sugammadex, compared with neostigmine, significantly hastened TOF recovery in geriatric patients undergoing spine surgery in the prone position and provided better predictability of reversal from NMB and discharge from the OR. However, sugammadex did not improve the length of PACU stay or the time to the first ambulation. Geriatric patients could be safely kept paralyzed with rocuronium infusion to avoid coughing, bucking, hypoventilation, or extubation until the end of lumbar spine surgery in the prone position. Then, they can be adequately reversed with sugammadex after returning to the supine position when the airway could be easily controlled without prolonging extubation and recovery.
